# Performance Fatigability is Associated with Differential Gene Expression Patterns in Human Skeletal Muscle

**DOI:** 10.1093/geroni/igaf122.3637

**Published:** 2025-12-31

**Authors:** Emma Gay, Paul Coen, Rebecca Deek, Brenda Diergaarde, Samaneh Farsijani, Michael Jurczak, Yujia Qiao, Nancy Glynn

**Affiliations:** University of Pittsburgh, Pittsburgh, Pennsylvania, United States; AdventHealth, Orlando, Florida, United States; University of Pittsburgh, Pittsburgh, Pennsylvania, United States; University of Pittsburgh, Pittsburgh, Pennsylvania, United States; University of Pittsburgh, Pittsburgh, Pennsylvania, United States; University of Pittsburgh, Pittsburgh, Pennsylvania, United States; University of Pittsburgh, Pittsburgh, Pennsylvania, United States; University of Pittsburgh, Pittsburgh, Pennsylvania, United States

## Abstract

Performance fatigability, the degree one is limited by fatigue during a physical task, is a common trait among older adults and is associated with physical and cognitive function. Knowledge of the biological mechanisms underlying performance fatigability is limited; the skeletal muscle transcriptome may reveal important pathways to prevent or mitigate performance fatigability and promote longer healthspan. The accelerometry-based Pittsburgh Performance Fatigability Index (PPFI) quantified percentage of cadence decline (i.e., slowing down) during the usual-paced 400m walk (range 0-100%). We used available cross-sectional skeletal muscle RNAseq data from the Study of Muscle, Mobility and Aging (n = 724, 55.5% Women, 86.9% White) to identify differentially expressed genes in participants with performance fatigability (PPFI>0%, n = 455, 62.8%) and those without performance fatigability (PPFI=0%, n = 269, 37.2%). A total of 1,935 differentially expressed genes were identified (DESeq2; adjusted for age, sex, and batch, false discovery rate adjusted p-value < 0.05). Of these genes, 594 were down-regulated and 1,341 were up-regulated in participants with performance fatigability vs. those without. Gene set enrichment analysis identified up-regulation in those with performance fatigability in four key muscle-related pathways: collagen organization/processing, immune system activation/response, extracellular organization and muscle cell development/function pathways; no pathways were significantly enriched in the under-expressed genes. Upregulation of these four pathways is known to be associated with phenotypic changes in aging muscle that leads to loss of strength and function, all important for maintaining mobility in older age. Pharmaceutical or therapeutic interventions that improve muscle quality may also concomitantly reduce performance fatigability, its sequelae, and extend healthspan.

